# Supplementary material for: Yoga an effective strategy for self-management of stress-related problems and wellbeing during COVID19 lockdown: A cross-sectional study
Source: PLoS One. 2021 Feb 10;16(2):e0245214. doi: 10.1371/journal.pone.0245214 (PMC7875402; doi:10.1371/journal.pone.0245214)
Supplement: S1 Table — (DOCX) [file pone.0245214.s001.docx]

S1 Table. Correlations

|  |  | 1 | 2 | 3 | 4 | 5 | 6 | 7 | 8 | 9 | 10 | 11 | 12 | 13 | 14 | 15 | 16 | 17 | 18 | 19 | Mean | Std. Deviation |
| --- | --- | --- | --- | --- | --- | --- | --- | --- | --- | --- | --- | --- | --- | --- | --- | --- | --- | --- | --- | --- | --- | --- |
| 1 | Consequence(IP1) | 1 |  |  |  |  |  |  |  |  |  |  |  |  |  |  |  |  |  |  | 7.58 | 2.32 |
| 2 | Timeline (IP2) | .283^**^ | 1 |  |  |  |  |  |  |  |  |  |  |  |  |  |  |  |  |  | 5.31 | 2.29 |
| 3 | Personal control (IP3) | -0.012 | 0.064 | 1 |  |  |  |  |  |  |  |  |  |  |  |  |  |  |  |  | 5.71 | 2.75 |
| 4 | Treatment Control (IP4) | .107^**^ | 0.048 | .315^**^ | 1 |  |  |  |  |  |  |  |  |  |  |  |  |  |  |  | 6.48 | 2.71 |
| 5 | Identity (IP5) | .191^**^ | .191^**^ | .172^**^ | .175^**^ | 1 |  |  |  |  |  |  |  |  |  |  |  |  |  |  | 5.75 | 2.23 |
| 6 | Illness concern(IP6) | .385^**^ | .225^**^ | -0.048 | .142^**^ | .220^**^ | 1 |  |  |  |  |  |  |  |  |  |  |  |  |  | 7.72 | 2.66 |
| 7 | Coherence/understanding (IP7) | 0.074 | 0.077 | .268^**^ | .158^**^ | .201^**^ | .261^**^ | 1 |  |  |  |  |  |  |  |  |  |  |  |  | 7.36 | 2.29 |
| 8 | Emotional representation (IP8) | .163^**^ | .171^**^ | -.143^**^ | .162^**^ | .133^**^ | .407^**^ | .117^**^ | 1 |  |  |  |  |  |  |  |  |  |  |  | 5.26 | 3.11 |
| 9 | Risk perception (IP10) | .154^**^ | .122^**^ | -.172^**^ | -0.002 | .216^**^ | .267^**^ | 0.057 | .234^**^ | 1 |  |  |  |  |  |  |  |  |  |  | 6.09 | 2.68 |
| 10 | Risk perception (IP11) | .136^**^ | .156^**^ | -.193^**^ | -0.016 | .165^**^ | .293^**^ | 0.037 | .221^**^ | .734^**^ | 1 |  |  |  |  |  |  |  |  |  | 5.38 | 2.61 |
| 11 | Personal preventative control(IP12) | 0.031 | .083^*^ | .361^**^ | .221^**^ | .156^**^ | 0.067 | .319^**^ | -0.015 | -.124^**^ | -.169^**^ | 1 |  |  |  |  |  |  |  |  | 6.83 | 2.47 |
| 12 | Depression (DASS-D) | 0.052 | 0.028 | -.135^**^ | -0.017 | 0.033 | 0.005 | -.086^*^ | .197^**^ | .148^**^ | .130^**^ | -.151^**^ | 1 |  |  |  |  |  |  |  | 0.86 | 0.66 |
| 13 | Anxiety (DASS-A) | 0.010 | .117^**^ | -0.041 | 0.050 | .104^**^ | 0.043 | -0.006 | .301^**^ | .156^**^ | .168^**^ | -0.076 | .430^**^ | 1 |  |  |  |  |  |  | 0.42 | 0.52 |
| 14 | Stress(DASS-S) | .079^*^ | 0.036 | -.116^**^ | -0.011 | .090^*^ | 0.063 | -0.071 | .182^**^ | .091^*^ | .111^**^ | -.088^*^ | .540^**^ | .533^**^ | 1 |  |  |  |  |  | 0.85 | 0.62 |
| 15 | Peace of Mind (POMS) | 0.006 | 0.022 | .303^**^ | .194^**^ | 0.071 | -0.011 | .278^**^ | -.203^**^ | -.118^**^ | -.138^**^ | .269^**^ | -.473^**^ | -.371^**^ | -.374^**^ | 1 |  |  |  |  | 3.30 | 1.03 |
| 16 | Wellbeing (SGWB) | 0.039 | 0.016 | .318^**^ | .191^**^ | .112^**^ | 0.029 | .288^**^ | -.156^**^ | -.101^*^ | -.130^**^ | .312^**^ | -.460^**^ | -.278^**^ | -.259^**^ | .734^**^ | 1 |  |  |  | 3.52 | 0.83 |
| 17 | Resilience(BRS) | 0.022 | 0.022 | 0.044 | -0.064 | 0.038 | .134^**^ | .080^*^ | -0.058 | 0.020 | 0.070 | .136^**^ | -.186^**^ | -.195^**^ | -.102^*^ | .173^**^ | .199^**^ | 1 |  |  | 3.13 | 0.70 |
| 18 | Emotion regulation-Cognitive Appraisal (ERQ-C) | 0.068 | 0.057 | .239^**^ | .179^**^ | .091^*^ | .121^**^ | .300^**^ | 0.006 | -0.019 | 0.003 | .308^**^ | -.202^**^ | -0.066 | -.097^*^ | .378^**^ | .448^**^ | .166^**^ | 1 |  | 5.02 | 1.15 |
| 19 | Emotion regulation-(Expressive Suppression (ERQ-E) | 0.051 | .087^*^ | .148^**^ | .155^**^ | 0.073 | .079^*^ | .130^**^ | .102^**^ | 0.038 | 0.028 | .147^**^ | .084^*^ | 0.054 | -0.033 | 0.074 | .085^*^ | -0.052 | .259^**^ | 1 | 4.50 | 1.34 |
